# Case of Amalgamated Placenta

**Published:** 1875-03

**Authors:** 


### CASE OF AMALGAMATED PLACENTA.

Dr. E. A. Eakin (*Lancet and Observer*) reports a case where, after waiting forty-eight hours after the delivery of the child for the extrusion of the after-birth, he anæsthetized his patient and found an hour-glass contraction. After overcoming this and getting his hand into the womb, he says, "I could find no beginning to the placenta, and very little of a mass to correspond. * * * * I accordingly began to pick into the mass, and having made an opening with difficulty, I succeeded in bringing a piece away about one and a-half by three-fourths of an inch in size. This was as perfectly organized as the tissue of the womb itself, with no decomposition whatever. I tried to remove more, but could not succeed." The case was then left to Nature, and the patient supported.

The second day after confinement the patient had two chills, followed by a pulse of 150, and feeble; headache, coated tongue, tenderness over the womb, and no hemorrhage.

During the next two days the pulse ranged from 110 to 126; skin became moist, tongue cleaned off, and tenderness over the bowels and womb was noted.

On the sixth day the pulse was 140, full and bounding; skin moist and cool, discharge scanty and offensive, and facies cadaveric.

On the eigth day, "threads" of placenta were expelled "about the size of a large twine, two inches long," and few in number.

The ninth day the lady was considerably jaundiced, and had a "fainting spell," which were attributed to biliary derangement.

On the tenth day the pulse was "114, full and regular; general appearance much improved; skin clear, moist and cool; no tenderness whatever," and the lochia became "more natural and abundant, with slight hemorrhagic tendency." The patient now gradually improved until the fourteenth day, when there occurred complete paralysis of the left side. "Pulse 80, and very intermittent," which only lasted a few hours. Recovery was gradual from this to the twenty-seventh day after delivery, when she was discharged.

The Doctor closes with the following remarks:

"There are a few points of peculiar interest in this case which I propose to mention, requesting comments from the profession.

"I have had a great many cases of *retained* and *adhered* placenta; but I prefer to call this a case of '*amalgamated* placenta,' if we recognize such a name, and I believe we do, as I find two cases mentioned in Churchill's Obstetrics. In mention of this case to an aged and very reputable teacher of this subject, he said he regarded it simply an *adhered* placenta. Now, as much as I regard the opinions of those having given the subject their life-study, I beg to differ in this opinion. I believe we should class as 'adherent placentas,' those *adhering* and *finally* coming away; as '*amalgamated*,' those *perfectly* adherent, having no points of detachment, and *never coming away*.

"Now, I believe this case in point to be an 'amalgamated' placenta, for these reasons: 1. It was much smaller than I ever knew one at 'full period'; 2. I could not find a beginning to membrane, or even origin of the fibers of the placenta; 3. When I succeeded in opening it in its center, I could only bring away what naturally twisted off by the greatest effort, and it was perfectly organized tissue, firm as beef, the vessels appearing more like ordinary blood-vessels, and very contracted; and, 4. It *never* came away, there now being no discharge. I will here state the discharges were never much more than natural, and part of the time none.

"Another point in the patient is: During her fourth confinement the placenta adhered slightly; the fifth more; in the sixth still more, in fact remained two or three days, until we have the seventh case before us. Question: What are the scientific reasons for the gradual increase of adhesions?

"Again, the paralysis in this case was similar to the same trouble she had in her sixth confinement, and occurred the same number of days and—I think—hours after labor. Have you any explanations?

"One thing more and I close. In this lady's sixth confinement the lochia ceased, and all the symptoms of puerperal fever set in; and after trying every other means, I resorted to the iodide of potash, with bichloride of mercury, and upon the administration of a few doses the flow appeared, and she began to improve immediately. In her last confinement the discharge ceased several times, and it was readily re-established by the same treatment. And I would say, even since 1871, I have resorted to it in the cessation of the lochia, and always with success; sometimes, however, without the mercury."—*Missouri Medical Record.*

